# Effects of Cha-Cha Dance Training on Physical-Fitness-Related Indicators of Hearing-Impaired Students: A Randomized Controlled Trial

**DOI:** 10.3390/bioengineering10091106

**Published:** 2023-09-21

**Authors:** Han Li, Youngsuk Kim, Zhenqian Zhou, Xuan Qiu, Sukwon Kim

**Affiliations:** 1 Department of Physical Education, Yichun University, Yichun 336000, China; lihan5557@163.com; 2 Department of Physical Education, Anhui Normal University, Wuhu 241000, China; 3 Department of Physical Education, Jeonbuk National University, Jeonju 54896, Republic of Korea; ys43530@jbnu.ac.kr; 4 School of Artistic Sport, Hunan Agricultural University, Changsha 410000, China; zzq1983@hunau.edu.cn

**Keywords:** dance sports, cha-cha dance, hearing impaired, physical fitness, strength

## Abstract

(1) Background: The physical fitness (PF) of hearing-impaired students has always been an international research hotspot since hearing-impaired students have difficulty in social interactions such as exercise or fitness programs. Sports interventions are proven to improve the fitness levels of hearing-impaired students; however, few studies evaluating the influence of Cha-cha (a type of Dance sport) training on the PF levels of hearing-impaired students have been conducted. (2) Purpose: This study aimed to intervene in hearing-impaired children through 12 weeks of Cha-cha dance training, evaluating its effects on their PF-related indicators, thus providing a scientific experimental basis for hearing-impaired children to participate in dance exercises effectively. (3) Methods: Thirty students with hearing impairment were randomly divided into two groups, and there was no difference in PF indicators between the two groups. The Cha-cha dance training group (CTG, *n* = 15) regularly participated in 90-min Cha-cha dance classes five times a week and the intervention lasted a total of 12 weeks, while the control group (CONG, *n* = 15) lived a normal life (including school physical education classes). Related indicators of PF were measured before and after the intervention, and a two-way repeated-measures analysis of variance was performed. (4) Results: After training, the standing long jump (CONG: 1.556 ± 0.256 vs. CTG: 1.784 ± 0.328, *p* = 0.0136, ES = 0.8081), sit-and-reach (CONG: 21.467 ± 4.539 vs. CTG: 25.416 ± 5.048, *p* = 0.0328, ES = 0.8528), sit-ups (CONG: 13.867 ± 4.912 vs. CTG: 27.867 ± 6.833, *p* < 0.0001, ES = 2.4677) and jump rope (CONG: 52.467 ± 29.691 vs. CTG: 68.600 ± 21.320, *p* = 0.0067, ES = 0.6547) scores showed significant differences. (5) Conclusions: After 12 weeks of Cha-cha dance training for hearing-impaired students, the PF level of hearing-impaired students in lower-body strength, flexibility, core strength, and cardiorespiratory endurance were effectively improved; however, there was no significant change in body shape, upper-body strength, vital capacity, and speed ability.

## 1. Introduction

Childhood hearing impairment is a significant public health problem in the world, which is associated with long-term academic and communicative difficulties [1,2]. It has been estimated that there are 360 million deaf or hard-of-hearing persons (over 5% of the world’s population) internationally [3]. The incidence of moderate-to-severe hearing loss in children is extremely high, with approximately 1–6 out of 1000 [4,5,6,7]. Hearing is an important sense that enables individuals to interpret their environment and communicate with others [8]. Hearing impairment is a disability that has a significant impact on a child’s cognitive, emotional, and social development/socialization, affecting the entire life of the hearing-impaired person [8,9,10].

Throughout the previous research related to physical fitness (PF), it is well-known that the social participation of hearing-impaired persons is lower than that of normal persons, and physical factors such as muscle strength, explosive power, coordination, speed, and balance are lower than normal persons [11,12].

The organs related to hearing and the vestibular system develop and function together. The receptors of these organs are in the inner ear. If the cochlea or semicircular canals are damaged, it leads to an abnormal vestibular system related to postural balance [13,14]. Due to these properties, persons with hearing impairment have slow responses to rapid or complex movements, thus leading to postural imbalance [15]. The imbalance can negatively affect other motor abilities, such as sensory integration, dynamic movement, and synergy with visual senses [16].

In addition, restricted physical activity impairs health-related PF and motor development, such as walking, running, and high jumping, and leads to the loss of basic abilities needed for daily living [17]. Particular functions of body control, such as balance and coordination, may also be adversely affected in hearing-impaired students [17]. Therefore, it is necessary to design an exercise program that considers the field of hearing-impaired groups and the decline of auditory function and physical factors. Thus, the program Is suggested to aid communication, proprioceptor development, and balance.

Physical training programs improve motor skills, balance, and health-related lifestyle qualities in hearing-impaired students [18], at the same time, they can also help hearing-impaired children improve their muscles and nervous systems, psychological responses, and motor development [19]. Dance sports are already popular around the world. No matter how it is used—such as the training of sports dance for children and teenagers, the special courses of sports dance in universities, or learning sports dance for entertainment and fitness by the elderly—sports dance is suitable for fitness improvement aerobic exercises for men, women, and children, and is also an art project for cultivating one’s own temperament and promoting psychological wellbeing [20,21]. Numerous studies have shown that long-term Dance sports training helps develop stamina, speed, flexibility, balance, and many other physical qualities [21,22,23]. As a dance category of Dance sports, Cha-cha has the main technical characteristics of the control technique of body posture, the fast-moving and rhythmic springing technique of the center of gravity, and the strong twisting technique of the hip, waist, and abdomen. These movements require the inner thigh muscles to exert force, thus, driving the entire lower limb joints, making the dance look more explosive, and performing better movement skills [24].

Numerous studies have shown improved balance ability [25], sensitivity [26], strength capacity [27], coordination [28], and cardiorespiratory endurance [29] in hearing-impaired students using dance-related programs that combine various dance techniques; however, there is still a great lack of research on the intervention of Cha-cha dance (a type of Dance sport) to improve the PF level of hearing-impaired students. Therefore, the purpose of this study is to intervene in hearing-impaired children through 12 weeks of Cha-cha dance training, evaluating its impact on the PF-related indicators of hearing-impaired children, thus providing a scientific experimental basis for the participation of hearing-impaired children in more appropriate and effective dance exercises.

## 2. Materials and Methods

### 2.1. Subjects

**Figure 1:**
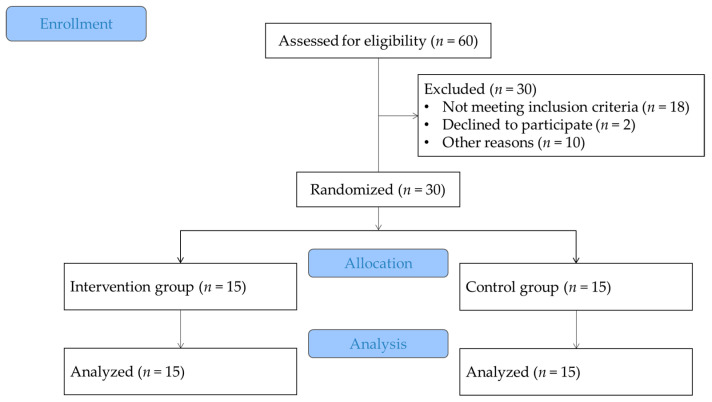
Flowchart of the present study; subject, screening, allocation, intervention, and analysis.

Thirty hearing-impaired students from a special school in Yichun, China, participated in the training course for 12 weeks (Table 1). They all signed an informed consent form approved by the University Ethics Committee (JBNU2022-01-004-002) and the assent process for participants was included during it. Participants were randomly assigned to the Cha-cha training group (CTG), and the control group (CONG) (Figure 1). The inclusion criteria for participants were as follows: (1) the age conforms to the definition of a student (10–18 years old); (2) binaural hearing threshold ≤ 55 dB; (3) there was no habit of participating in regular sports for a long time; (4) there was no structural injury or disease of the extremities in the past year; (5) mental health—no mental illness; (6) willing to sign the participation agreement (including the safety agreement). In the end, 48 preliminary subjects who met the above six criteria were found out of 60 valid questionnaires to enter the precise screening. The screening content mainly included two aspects: FMS functional exercise test and body mass data [30] were used to ensure PF and safety; and the basic body indicators. There was no statistically significant difference in FMS body function test scores and body mass data between the two groups (*p* = 0.61) (Table 1).

### 2.2. Intervention

In this study, CTG trained for 45 mins five times a week, with a 5-min rest in between. CTG starts at an elementary level and is trained progressively to an intermediate level and then to an advanced level (Table 2). The target heart rate is set at 50 to 60% of the HR max and approximately 75 to 90 bpm throughout the process, not exceeding 70% of the HR max (approximately 105 bpm). A Polar watch (Polar Electro Inc., Bethpage, NY, USA) was used to monitor the heart rate (Table 3). CONG did not perform any exercise interventions, only daily life (including normal school physical education).

### 2.3. Dependent Variable and Measurements

Within the scope of the physical fitness test battery, physical characteristics (BMI), performance characteristics (strength, endurance, speed, flexibility), and physiological characteristics (Vital capacity) were measured.

#### 2.3.1. BMI

Participants’ height and weight were measured using a portable instrument (GMCS-IV; Jianmin, Beijing, China) to reflect their anthropometric characteristics. Age was measured in years. Height was measured barefoot or with socks using the height scale on the bascule with a sensitivity level of 0.01 cm. Body weight was measured with shorts on the bascule with 0.01 kg sensitivity [31]. BMI was measured by height and weight levels with the formula: BMI = (kg)/m^2^ [32].

#### 2.3.2. Standing Long Jump

The standing long jump method is used to test the explosive power of hearing-impaired students on a flat plastic track, and the students wear comfortable sports shoes. After the long jump was performed using both feet—without acquiring speed from the standing position—the interval between the jumping point to the last point the athlete leaves a trace at was measured in m. The test was applied three times to participants and the best result was recorded.

#### 2.3.3. Grip Strength

Measurements of hand grip strength of hearing-impaired participants were made by the Jamar hand dynamometer (Lafayette Instrument Company, Lafayette, IN, USA). Participants were asked to bend the dominant arm while standing and to grasp the dynamometer with all their strength. After three repetitions, best result was recorded in kg [33].

#### 2.3.4. Sit-and-Reach

The sit-and-reach test was carried out by a seat-forward flexion tester (GMCS-IV; Jianmin, Beijing, China) to assess flexibility. Participants warmed up before the test. For the measurement, the subject took off their shoes, sat down on the measuring table, straightened their knees, and pushed the ruler forward. The record was measured when the subject flexed, reached the farthest point, and stopped for 2 s, and the highest record out of a total of three trials was selected, measured in cm [34].

#### 2.3.5. Vital Capacity

The most important aspect of a spirometer is forced vital capacity (FVC), which is the maximum volume of air that can be exhaled with maximum force during maximum inspiration. The FVC maneuver is divided into three distinct phases, as follows: (1) maximum inhalation; (2) exhalation “explosion”; and (3) continuous full exhalation until the end of the test. The test was performed three times and the maximum result was taken; the measurement was recorded in milliliters [35].

#### 2.3.6. Sit-Ups

Sit-ups are simple tests that can accurately assess abdominal strength without the use of special equipment. Before testing, the subject laid down with knees bent, feet on the ground, and hands placed on their chest. The assistant held the subject’s feet on the floor. During the 1-min test, the subject’s back must touch the base, and we measured the number of times elbows touched the knees while the upper body was raised. This test was measured only once to exclude fatigue effects [36].

#### 2.3.7. Fifty-Meter Dash

In this study, the 50-m speed test was used to evaluate the speed of the subjects. After warming up, the subject was asked to run 50 m as fast as possible. This test was performed once, the time was calculated with a stopwatch, and the measurement was recorded in s [37].

#### 2.3.8. Jump Rope

Jump-rope exercise tests are widely used to assess cardiorespiratory endurance [38] and flexibility [39]. In this study, jumping rope was used to perform cardiorespiratory endurance testing and the number of successful jumps in one minute was recorded. The test was only measured once to exclude the effect of fatigue.

### 2.4. Statistical Methods

Data are expressed as mean ± SD and statistical significance was accepted as *p* < 0.05. Variables were analyzed using two-way repeated-measures ANOVA, followed by Šidák’s multiple comparisons; the factors were intervention conditions (CONG/LTG) and time (pre-/post-intervention). An unpaired *t*-test was performed to analyze the pre-intervention variables. Cohen’s d effect size (ES) was reported (ES > 0.2, small; >0.5, moderate; >0.8, large) when statistical differences were found between conditions. Statistical analyses were performed using GraphPad Prism 9.2.0 (GraphPad Software, La Jolla, CA, USA).

## 3. Results

### 3.1. Comparison of Baseline Features before Training

In all measures of fitness variables, there was no significant difference between the CTG and the CONG (*p* > 0.05) (Table 4).

### 3.2. Effect of Intervention

The standing long jump results are depicted in Table 5. Following the ANOVA, a main time effect was found (*p* < 0.01). Standing long jump scores were similar at baseline and significantly improved after training. The standing long jump performance of the CTG is higher than that of the CONG after training (CONG: 1.556 ± 0.256 vs. CTG: 1.784 ± 0.328, *p* = 0.0136, ES = 0.8081). At the same time, it was found that through Cha-cha dance training, there was a significant interaction between time × group (*p* < 0.05).

Sit-and-reach results are depicted in Table 5. Following the ANOVA, a main time effect was found (*p* < 0.01). Sit-and-reach scores were similar at baseline and significantly improved after training. The sit-and-reach performance of the CTG is higher than that of the CONG after training (CONG: 21.467 ± 4.539 vs. CTG: 25.416 ± 5.048, *p* = 0.0328, ES = 0.8528). At the same time, it was found that through Cha-cha Dance training, there was a significant interaction between time × group (*p* < 0.05).

Sit-up results are depicted in Table 5. Following the ANOVA, a main time effect was found (*p* < 0.01). Sit-up scores were similar at baseline and significantly improved after training. The sit-up performance of the CTG is higher than that of the CONG after training (CONG: 13.867 ± 4.912 vs. CTG: 27.867 ± 6.833, *p* < 0.0001, ES = 2.4677). At the same time, it was found that through Cha-cha Dance training, there was a main effect for the group (*p* < 0.01) as well as a significant interaction between time × group (*p* < 0.05).

The jump rope results are depicted in Table 5. Following the ANOVA, a main time effect was found (*p* < 0.01). The jump rope scores were similar at baseline and significantly improved after training. The jump rope performance of the CTG is higher than that of the CONG after training (CONG: 52.467 ± 29.691 vs. CTG: 68.600 ± 21.320, *p* = 0.0067, ES = 0.6547).

However, through the ANOVA, it was found that the four test indicators of BMI, grip strength, vital capacity, and the 50-m dash had no significant effects after the experiment between the two groups. In summary, after 12 weeks of Cha-cha dance training intervention, hearing-impaired children have significantly improved their lower-body strength, flexibility, core strength, and cardiorespiratory endurance. Although there was no significant difference in body shape, upper-body strength, vital capacity, and speed ability before and after the experiment, they improved a little over time.

## 4. Discussion

This study assessed whether 12 weeks of Cha-cha dance training helped improve PF in hearing-impaired students. Results of this study showed that 12 weeks of Cha-cha dance effectively improved lower-body strength, flexibility, core strength, and cardiorespiratory endurance in hearing-impaired students. At the same time, upper-body strength, lung capacity, and speed ability also improved over time. This finding is consistent with CAGLAR, et al. [40], Mulya [41], Özdemir, et al. [42], and Mahasuran [43].

Hearing loss usually appears in childhood, most of these hearing-impaired persons have delayed motor function development from an early age [44]. Previous research has shown that hearing-impaired children have lower levels of physical fitness than healthy children of the same age, as evidenced by delayed motor development, postural failure, balance failure, poor muscle strength, and social adjustment problems [45].

Latin dance is a coordinated exercise of sport and music, which can improve physical and mental health [46] and has a positive effect on weight control and fat loss [47]. The Cha-cha dance is characterized by the number of steps per unit of time, the movement is mostly symmetrical, the muscle activity affects the whole body, and the center of gravity moves fast [21]. The findings from the present study suggested that these characteristics of the Cha-cha dance had effects on improving the neuromuscular ability, the sensory integration ability, and the ability to control the muscles of hearing-impaired students, thus effectively improving PF-related indicators.

In terms of the standing long jump in the present study, the change in lift before and after the training intervention was evident. The standing long jump is an important means to test the maximum power and explosive power of lower limbs. During the Cha-cha dance practice, the continuous support of the lower-limb muscles is needed to ensure the balance of the body’s center of gravity, which is also the main reason for the improvement of the lower-limb power of hearing-impaired children [23]. Motor skills play an important role in dance performance, which is closely related to coordination, power, and speed [48,49]. In addition, improvement of lower-extremity muscle power is closely related to improvement of balance ability [50]; therefore, the improvement in standing long jump ability in this study suggests that Cha-cha dance training is effective in improving lower-extremity muscle power and the balance ability of students with hearing impairment.

In terms of sit-and-reach, the change in lift before and after the training intervention was evident. Dance needs to be characterized by a graceful stature and line, much of which is due to flexibility [51]. Cha-cha dance requires dancers to reflect the beauty of the dance by elongating the entire body line, including upper-body lines and lower-body lines. Repeated practice for a long time would effectively improve flexibility. In addition, dance therapy can improve flexibility and reduce muscle tension [52]. Flexibility can affect the range of motion and posture of joints, which is an essential factor in developing a balanced physique or physical strength in growing children [53]. As a result, it was found that improving the sit-and-reach ability after training in Cha-cha dance is effectively improving children’s flexibility and forming a well-balanced physique.

In terms of sit-ups, 12 weeks of Cha-cha training showed significant changes in its results, and the sit-up test assessed not only abdominal muscular endurance but also static strength. During the Cha-cha dance training, the subjects were required to keep their upper body upright and their lower-body muscles relaxed, and the connected abdomen (core strength area) should always maintain a certain degree of muscle tension to maintain the stability of the whole body during dance movements. Cha-cha dance is characterized by various movements of the hip joint and pelvis [54]. This characteristic of the dance twist of the upper body and increasing and releasing pressure in the lower body helps improve muscle support in the lower back, abdomen, and lower body [55]. The result from the sit-ups suggested that these movement characteristics of the Cha-cha dance had effects on improving the core strength of hearing-impaired students.

In terms of the jump rope, the changes before and after training are significantly improved. The jump rope can evaluate cardiorespiratory endurance, flexibility, and lower-body strength. Studies have shown that using a skipping rope is positively correlated with cardiorespiratory endurance and the maximum strength of the lower body [56]. According to AAHPER, it was emphasized that cardiorespiratory capacity is more important for individuals with disabilities because they show low physical fitness levels [57]. Among the five types of Latin dance, the exercise intensity per unit of time for the Cha-cha dance is on the high side [58] but the intensity of the training of the Cha-cha dance in this study corresponds to low-intensity exercise based on heart rate. Low-intensity aerobic exercise is an effective exercise method for improving cardiorespiratory endurance in children with disabilities [59]. The result of the present study’s improvement in jumping rope ability suggests that 12 weeks of low-intensity Cha-cha dance training is the leading cause of improving the cardiorespiratory endurance of hearing-impaired students.

However, in terms of BMI, grip strength, vital capacity, the 50-m dash, although there are no significant differences in the changes before and after training, there are certain improvements over time; with only 45 min of physical activity 5 times a week, the amount of exercise was insufficient to induce changes in these parameters. In addition, the study’s participants were hearing-impaired children who had never experienced dance-related movement practice, and it took more time to master the dance moves than for ordinary persons. The level of mastery of dance is different, the intensity of exercise is different, and the physiological stimulation that follows will also be different [60]. Therefore, more adaptation time is required from completing the correct action to increasing the exercise intensity; thus, the overall exercise intensity may be weakened during the exercise program and unable to cause appropriate changes in these indicators.

## 5. Conclusions

After 12 weeks of Cha-cha dance training for hearing-impaired students, the PF of hearing-impaired students in lower-body strength, flexibility, core strength, and cardiorespiratory endurance have been effectively improved.

Therefore, Cha-cha dance is an appropriate exercise program for hearing-impaired persons with reduced physical activity due to hearing impairment that can improve motor function execution and serve as a campus physical education course. In this study, there were fewer subjects during the experiment, and there are certain limitations in the literature on Cha-cha dance training; therefore, this experiment will conduct a follow-up in-depth study on the intervention of Cha-cha dance on the PF of hearing-impaired students and explore its specific mechanisms of action.

## Figures and Tables

**Table 1:** Basic information of experimental subjects.

Group	Age	Height (cm)	Weight (kg)	FMS (Score)
CONG (*n* = 15)	14.889 ± 2.208	151.81 ± 2.21	36.96 ± 1.21	10.45 ± 1.79
CTG (*n* = 15)	15.333 ± 2.121	152.25 ± 1.98	36.58 ± 2.59	10.15 ± 1.90
*p* value	0.66	0.74	0.89	0.61

**Table 2:** Detailed Action Training Schedule.

Time	First Class	Second Class	Third Class
Week 1	Basic steps learned: (1) Basic movement (2) New York (3) Time step	1. Review of basic steps 2. Basic steps learned: (1) Hockey stick (2) Forward Lock (3) Natural top	1. Review of basic steps 2. Basic steps learned: (1) Back Lock (2) Hand to Hand (3) Underarm turn to Left
Week 2	1. Review of basic steps 2. Basic steps learned: (1) Open hip twist Turn (2) 1–5 Close Basic movement	1. Review of basic steps 2. Basic steps learned: (1) Basic movement with 1/4 (2) Natural Top	1. Review of basic steps 2. Basic steps learned: (1) Cuba rocks (2) Syncopate New York (3) Spot Turn
Week 3–7	Single basic step combination exercise	Music rhythm single basic step combination practice	Male and female doubles exercise
Week 8–12	Music rhythm male and female doubles exercise	Male and female doubles ready for competition stage exercise	Male and female doubles competition stage

**Table 3:** Exercise Load Control in CTG (heart rate).

Learning Stage	Period	HR (Times/Min)
Basic stage	Weeks 1–2	70.39 ± 12.59
Consolidation stage	Weeks 3–7	77.68 ± 17.72
Improved stage	Weeks 8–12	86.47 ± 14.59

**Table 4:** Students’ PF characteristics during the baseline test.

Variable	CONG (*n* = 15)	CTG (*n* = 15)	*p*
BMI (kg/m^2^)	18.011 ± 4.199	17.908 ± 3.939	0.95
Standing long jump (m)	1.483 ± 0.255	1.467 ± 0.308	0.88
Grip strength (kg)	22.723 ± 4.943	22.627 ± 3.604	0.95
Sit-and-reach (cm)	15.400 ± 3.776	15.067 ± 5.271	0.84
Vital capacity (mL)	1914.467 ± 482.705	1960.200 ± 366.773	0.77
Sit-ups (times/min)	15.400 ± 3.776	15.067 ± 5.271	0.84
Jump rope (times/min)	49.133 ± 28.028	46.800 ± 18.253	0.79
Fifty-meter dash (s)	11.536 ± 2.352	11.580 ± 1.841	0.95

**Table 5:** Results of PF indicators pre- and post-intervention.

Variables	Groups	Pre-Test	Post-Test
BMI (kg/m^2^)	CONG	18.011 ± 4.199	18.271 ± 3.454
	CTG	17.908 ± 3.939	18.444 ± 3.726
Standing long jump (m)	CONG	1.483 ± 0.255	1.556 ± 0.256
	CTG	1.467 ± 0.308	1.784 ± 0.328 *
Grip strength (kg)	CONG	22.723 ± 4.943	22.265 ± 4.857
	CTG	22.627 ± 3.604	23.822 ± 5.615
Sit-and-reach (cm)	CONG	21.600 ± 6.858	21.467 ± 4.539
	CTG	20.360 ± 6.170	25.416 ± 5.048 *
Vital capacity (mL)	CONG	1914.467 ± 482.705	1877.800 ± 449.235
	CTG	1960.200 ± 366.773	2118.667 ± 334.008
Sit-ups (times/min)	CONG	15.400 ± 3.776	13.867 ± 4.912
	CTG	15.067 ± 5.271	27.867 ± 6.833 **
Jump rope (times/min)	CONG	49.133 ± 28.028	52.467 ± 29.691
	CTG	46.800 ± 18.253	68.600 ± 21.320 **
Fifty-meter dash (s)	CONG	11.536 ± 2.352	11.732 ± 2.389
	CTG	11.580 ± 1.841	10.479 ± 1.840

CONG, normal life with no intervention; CTG, Cha-cha dance training five times per week, each class lasted for 45 min for 12 weeks. Two-way repeated measures ANOVA with Šidák’s post hoc test for all. Data are presented as the mean ± SD (*n* = 30). * *p* < 0.05 vs. CONG; ** *p* < 0.01 vs. CONG.

## Data Availability

All data generated or analyzed during this study are included in this published article.
